# Association between Dietary Inflammatory Index, C-Reactive Protein and Metabolic Syndrome: A Cross-Sectional Study

**DOI:** 10.3390/nu10070831

**Published:** 2018-06-27

**Authors:** Zhongxia Ren, Ai Zhao, Yan Wang, Liping Meng, Ignatius Man-Yau Szeto, Ting Li, Huiting Gong, Zixing Tian, Yumei Zhang, Peiyu Wang

**Affiliations:** 1 Department of Nutrition and Food Hygiene, School of Public Health, Peking University Health Science Center, Beijing 100191, China; renzhongxia@bjmu.edu.cn (Z.R.); huitinggong@163.com (H.G.); tianzx129@126.com (Z.T.); 2 Department of Social Science and Health Education, School of Public Health, Peking University Health Science Center, Beijing 100191, China; aizhao@bjmu.edu.cn (A.Z.); wpeiyu@bjmu.edu.cn (P.W.); 3 Inner Mongolia Dairy Technology Research Institute Co. Ltd., Hohhot 010110, China; wangyan0@yili.com (Y.W.); mengliping@yili.com (L.M.); szeto@yili.com (I.M.-Y.S.); liting2012@yili.com (T.L.); 4 Yili Innovation Center, Inner Mongolia Yili Industrial Group Co. Ltd., Hohhot 010110, China

**Keywords:** dietary inflammatory index, C-reactive protein, metabolic syndrome

## Abstract

Increased prevalence of metabolic syndrome (MetS) has become a global major public health problem. Chronic low-grade inflammation associated with diet was found to play an import role in the development of MetS, although further studies are needed. The main purpose of this study was to explore the association between the dietary inflammatory index (DII), C-reactive protein (CRP) as a sign of inflammation status, and MetS. A total of 1712 participants from eight cities in China were included. Sociodemographic and health-related information was collected by a self-administrated questionnaire. Anthropometric information and fasting blood samples were collected for identification of MetS. DII scores were computed based on one time 24-h dietary recall. No significant association between MetS and DII was observed except for the blood pressure component of MetS (OR _T3 versus T1_ = 1.40; 95% CI: 1.03 to 1.89). A significant increased prevalence for MetS was observed for higher CRP (OR = 1.66; 95% CI: 1.26 to 2.18), as well as four out of five of MetS components. In stratified analyses by sex, the associations between DII/CRP and MetS among women, but not men, are comparable to the whole sample. In addition, Both the 2nd and 3rd tertile of the DII had a higher CRP level (β-Coefficients _T2 versus T1_ = 0.086, 95% CI: 0.004 to 0.167; β-Coefficients _T3 versus T1_ = 0.145, 95% CI: 0.045 to 0.245) among subjects with MetS. Participants with higher DII scores reported a higher degree of “*Shanghuo*” (*p* = 0.007), which is a traditional concept characterized by “redness, swelling, fever and pain” in Chinese Medicine. This study suggested a close association between CRP and MetS, while the association between the DII and MetS was limited. DII was only specifically associated with CRP at a higher level among participants with MetS.

## 1. Introduction

The metabolic syndrome (MetS), defined as a complex of risk factors including elevated blood glucose levels, elevated triglyceride levels, reduced high-density lipoprotein cholesterol (HDL-c) levels, raised blood pressure, and obesity, has become a global public health problem [1]. Although the average figures remain difficult to evaluate due to the varying definitions and population distribution, the prevalence of MetS varies between 20% and 45% according to epidemiological studies from different countries [2]. The situation in China is not optimistic as a recent study reported that the average prevalence of MetS was 33.9% (31.0% in men and 36.8% in women) for 31 provinces of China in 2010 [3].

Even though researchers have proposed that insulin resistance played a causative role and central obesity was a highly possible early step in the development of MetS, the cause of MetS remains unclear to us [4]. Recent studies implicated that inflammation, especially chronic low-grade inflammation, might play an even greater role [5]. One possible mechanism is that the growth of adipose tissue and infiltration of immune cells lead to the increase of pro-inflammatory adipokines such as tumor necrosis factor alpha (TNF-α), C-reactive protein (CRP), and interleukin-6 (IL-6) [6,7,8,9], which cause increased insulin resistance from insulin-sensitive tissues by decreasing insulin signaling [10,11]. In addition, other components of MetS, such as dyslipidemia, glucose intolerance and hypertension were also found to be co-mechanisms with inflammation [5]. MetS raises major public health concerns as it is a known risk factor of cardiovascular diseases, in which immune mechanisms interact with metabolic risk factors to dominate and accelerate progression of lesions [12].

Diet has been demonstrated as an important lifestyle factor associated with MetS. Most previous studies were mainly concerned with the over nutrition of unhealthy dietary patterns, especially with high consumption of fat (energy-dense), which causes the imbalance of energy [13,14]. In addition, epidemiologic and clinical studies focused on the effects of diet on inflammation [15]. For example, higher consumption of meat-instant foods (rich in animal protein, saturated fat, sweets, sodium and food additives) was found to be significantly associated with CRP and MetS [16]. In a dietary study of individuals with MetS, the intake of saturated fats was associated with IL-1 Ra [17].

To improve the specificity for inflammation of dietary scores, the Dietary Inflammatory Index (DII) was first developed in 2009 [18] and then updated in 2014 [19] in order to evaluate the inflammatory potential of diet on a continuum. Two components will be taken into account in the evaluation of DII: statistically inflammatory potential and personal intake of specific food parameters, both of which can represent important sources of variability [20]. The relevance of the DII scores in the association between inflammation and cardio-metabolic diseases such as cardiovascular disease (CVD), MetS, and mortality has been critically reviewed and the DII seems to be a useful tool [20,21]. However, to date, no such studies have been performed in China.

In addition, from the perspective of traditional Chinese medicine (TCM), it is widely believed that the status of *Shanghuo* of the human body is closely related to diet, which in turn is related to health status. *Shanghuo*, characterized by “redness, swelling, fever, and pain”, usually occurs in infectious diseases especially in the middle and late period, and also exists in some non-infectious diseases such as autoimmune or endocrine diseases [22]. Considering that *Shanghuo* has symptoms similar to inflammation [23], in this study we also regard *Shanghuo* as a sign of inflammation. 

Thus, our main objective was to explore the association between the DII and the prevalence of MetS among adults in eight cities in China. The secondary objective was to investigate whether CRP, as one of the inflammatory factors, has a possible mediation effect between them. Further, we also aimed to evaluate whether the DII is simply associated with the self-reported frequency of *Shanghuo*.

## 2. Methods 

### 2.1. Subjects

This study is a sub-research of the Chinese Urban Adults Diet and Health study (CUADHS), which was conducted in eight cities in China from March to July 2016. The details of recruitment of subjects in this research have been described elsewhere [24]. Briefly, eight cities were purposively selected according to their geographical position and economy level. The convenience sampling method was then used to select two communities in each first-tier city (Beijing and Guangzhou) and one community in every non-first tier city (Chengdu, Chenzhou, Jilin, Lanzhou, Wuhu and Xuchang). Next, based on resident registration, a random sampling method was used to identify potential participants. Eligible subjects were ages 18–75 years old and natives of an urban area or having lived in urban areas for at least 1 year. A total of 1806 subjects were invited and 1739 of them were subsequently included with a response rate of 96.3%. At least 60, 60 and 50 residents were included for three age groups in every community: 18–44 years, 45–64 years, and >65 years, respectively. In accordance with the purpose of this study and its required data, 27 subjects were excluded due to missing responses in dietary records. Finally, the sample size of Beijing, Guangzhou, Chengdu, Chenzhou, Jilin, Lanzhou, Wuhu and Xuchang was 326, 326, 168, 173, 165, 175, 196 and 183, respectively.

### 2.2. Data Collection

Interviewers were trained to effectively collect baseline sociodemographic information using an interviewer-administered questionnaire. At the same time information was collected about self-reported health-related status such as physical activity status, smoking status and the frequency of “*Shanghuo*” (Never, Sometimes or Often/always). We used one-time 24-h dietary recall to collect information about daily food consumption with the help of a standard reference picture book, bowls, plates, and spoons. Nutrients intake was calculated according to a food composition database derived from the Chinese Food Composition (CFC) tables of 2004 and 2009 [25,26]. Standard protocols were used to take anthropometric measurements consisting of blood pressure, height, weight, and waist circumference. In addition, fasting blood samples were obtained to determine serum triglycerides, HDL-c, glucose and CRP by glycerol phosphate oxidase methods, directed methods, glucose oxidase methods and immunoturbidimetry, respectively, by a single qualified laboratory (Lawke Health Laboratory, Beijing, China).

### 2.3. Calculation of DII Scores

The details of the DII can be found elsewhere [19]. Briefly, a total of 1943 published studies were reviewed to assign an “inflammatory effect score” for 45 food parameters. A “world” mean and standard deviation was then established from 11 databases based on actual human consumption for each parameter. Raw food parameter intakes were calculated from the 24-h dietary recall record and were first standardized by subtracting the “world” mean and dividing by the “world” standard deviation, and then converted to percentile scores. The adjusted scores were multiplied by 2 and subtracted 1 to establish symmetrical distributions which centered on 0 and were bounded between −1 and +1. The centred percentile score for each parameter was then multiplied by its corresponding “inflammatory effect score”, and finally summed to obtain the overall DII score. Available food parameters in this study included: carbohydrate; protein; total fat; saturated, monounsaturated and polyunsaturated fatty acids; fibre; cholesterol; niacin; thiamine; riboflavin; folic acid; vitamins A, B6, B12, C and E; iron; magnesium; selenium; and zinc. The nutrient density method (intake per 1000 kcal of energy) was used to decrease the influence of the different energy intake among subjects [27].

### 2.4. Identification of the Metabolic Syndrome

MetS was identified as conforming to at least three of the following criteria according the 2009 Joint Interim Statement [1]: elevated waist circumference (≥90 cm for men, ≥80 cm for women), elevated triglycerides (≥1.7 mmol/L or drug treatment), reduced HDL-c (<1.0 mmol/L for men, <1.3 mmol/L for women or drug treatment), raised blood pressure (systolic blood pressure [SBP] ≥130 and/or diastolic blood pressure [DBP] ≥85 mm Hg or drug treatment), and elevated fasting glucose (glycaemia ≥5.6 mmol/L or drug treatment). A total of 1690 participants were eligible for determination of their MetS status in this study.

### 2.5. Ethics

This study was implemented in accordance with the Declaration of Helsinki. Ethical approval for research procedures was granted by the Medical Ethics Research Board of Peking University (No. IRB00001052-15059). Participation was voluntary and participants signed an informed consent document after being told the details of the study.

### 2.6. Statistical Analysis

Analyses were performed using IBM SPSS version 20.0 (International Business Machines Corporation, Armonk, NY, USA). DII scores were transformed to tertiles (Tertile 1 = −3.50 to 0.04; Tertile 2 = 0.05 to 1.11; Tertile 1 = 1.12 to 3.49). Normality for continuous data was tested before analysis. Values for serum CRP were log-transformed to improve normality. Descriptive statistics were presented as mean (standard deviation), P50th (P25th, P75th) or percentage. For single factor analysis, Chi-squared analysis or One-way ANOVA test were used for categorical or continuous confounding variables across tertiles of DII scores, respectively. Kruskal-Wallis tests were used for nutrients intakes across tertiles of DII. Binary logistic regression was carried out to explore the association between tertiles of DII scores/continuous CRP and dichotomous MetS outcomes, as well as dichotomous independent components of MetS. Linear regression was used to assess the relationship between tertiles of DII scores and serum CRP levels. In addition, the DII scores were analyzed as a continuous form. The multivariate analyses were also stratified by sex. The following confounding factors were included in the multivariate analysis according to baseline differences across DII tertiles with a *p* ≤ 0.20: age, gender, city, family monthly expenditure on food, smoking status and BMI. In addition, educational level was also included as a potential confounder according to an extensive review of related literature. The Chi-squared analysis was used to simply estimate the association between DII and the frequency of *Shanghuo*. The level of statistical significance in the study was set to *p* < 0.05.

Finally, for the purpose of optimizing the robustness of the statistical tests, we performed sensitivity analyses after removing participants who self-reported that they had changed their dietary habits. A total of 1319 subjects were eligible for inclusion in the supplementary analyses.

## 3. Results

A total of 1712 adults from eight cities in China were analyzed in this study, including 582 males and 1130 females, with an average age of 50.4 ± 17.4 years. The DII scores ranged from −3.50 (most anti-inflammatory) to 3.49 (most pro-inflammatory), and the mean ± standard deviation was 0.46 ± 1.16.

### 3.1. Baseline Characteristics 

Baseline characteristics of tertiles of DII scores are shown in Table 1. Compared with the lowest DII score tertile (T1), subjects with the highest DII scores (T3) were younger, and more of them were males, spent less on food and lived in south of China, especially in Chengdu, Chenzhou or Wuhu. 

Table 2 shows the macro- and micro-nutrients intakes according to tertiles of the DII. A higher DII score was significantly associated with higher intake of total fat and monounsaturated fatty acids, lower intake of carbohydrate, protein, polyunsaturated fatty acids, fiber, niacin, thiamine, riboflavin, folic acid, vitamins A, B6, B12, C, and E, iron, magnesium, selenium, and zinc (*p* < 0.05).

### 3.2. Incidence of MetS and DII/CRP

No significant association was observed between the prevalence of MetS or its individual components and DII, except for the blood pressure component for DII T3 compared to T1(OR _T3 versus T1_ = 1.40; 95% CI: 1.03 to 1.89), after considering confounding variables including age, gender, city, education level, family monthly expenditure on food, smoking status and BMI (Table 3). 

A significantly increased prevalence of MetS was observed for higher CRP (OR = 1.66; 95% CI: 1.26 to 2.18), as well as for four out of five MetS components as follows (Table 3): waist component (OR = 1.91; 95% CI: 1.36 to 2.66); HDL component (OR = 1.64; 95% CI: 1.27 to 2.11); triglycerides component (OR = 1.44; 95% CI: 1.12 to 1.85) and glucose component (OR = 1.73; 95% CI: 1.34 to 2.24).

The results of stratified analysis by sex are shown in Table 4. Similar associations were found among women: A significant association was observed for the blood pressure component of MetS for DII T3 compared to T1(OR _T3 versus T1_ = 1.72; 95% CI: 1.15 to 2.56); In addition, a significantly increased prevalence of MetS was observed for higher CRP (OR = 2.17; 95% CI: 1.49 to 3.15), as well as for four out of five of MetS components as follows: waist component (OR = 1.85; 95% CI: 1.20 to 2.88); HDL component (OR = 1.77; 95% CI: 1.31 to 2.38); triglycerides component (OR = 1.86; 95% CI: 1.33 to 2.60) and glucose component (OR = 1.69; 95% CI: 1.21 to 2.37). Among men, no significant association was observed except for the association between the glucose component and higher CRP (OR = 1.79; 95% CI: 1.17 to 2.73).

In supplementary analyses, conducted after removing participants who self-reported that they had changed their dietary habits, findings were similar (Table S1).

### 3.3. DII and CRP

No statistically significant mean differences for CRP were observed between the upper and lower DII tertiles among all subjects (Table 5), after adjusting for confounding variables. The same was true when using the continuous form of DII. 

When the data were stratified based on whether the participants had MetS, compared with T1, both the 2nd and 3rd tertiles of the DII had a higher CRP level (β-Coefficients _T2 versus T1_ = 0.086, 95% CI: 0.004–0.167; β-Coefficients _T3 versus T1_ = 0.145, 95% CI: 0.045–0.245) among subjects with MetS. The same was true when using the continuous form of DII (β-Coefficients = 0.040, 95% CI: 0.010–0.069). Among subjects without MetS, no significant differences were observed (Table 5). When the data were stratified by sex, no significant association between CRP and DII was observed in both men and women (Table S2).

In supplementary analyses, the differences were only significant when using the continuous form of DII among subjects with MetS (Table S3).

### 3.4. DII and the Frequency of “Shanghuo”

Among participants with highest DII scores (T3), the self-reported percentages of experiencing never, sometimes and often/always “*Shanghuo*” in the past six months were 11.8%, 59.5% and 28.7%, respectively. Among participants with median DII scores (T2), the corresponding percentages were 12.9%, 59.1% and 28.0%. Among T1, the corresponding percentages were 14.7%, 65.1% and 20.2%. Results of a Chi-squared analysis showed that the differences were significant (*p* = 0.007).

## 4. Discussion

**Figure 1:**
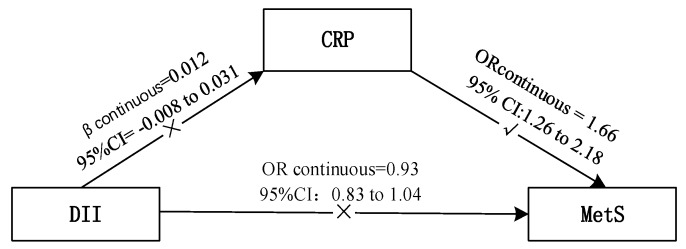
Association between DII, CRP and MetS. DII: dietary inflammatory index, CRP: C-reactive protein, MetS: metabolic syndrome.

In this study, no positive association was observed between the DII and MetS except for the blood pressure component. To further understand the association between them, we also investigated whether inflammation plays a role as mediator using CRP as a representative inflammatory marker (Figure 1). The results suggested that higher CRP was linked to increased prevalence of MetS and four out of five of its components. Among subjects with MetS, higher DII scores, reflecting a more pro-inflammatory diet, were associated with higher CRP, while in the overall sample the association was not significant.

To date, there have been two prospective studies and four cross-sectional studies investigating the inflammatory effects of diet on MetS. Similar with us, in three cross-sectional studies, higher DII scores were associated with only a few individual components of the MetS, such as glucose intolerance, greater diastolic blood pressure, and lower HDL-C levels, respectively [28,29,30]. One prospective study conducted in France suggested a significant association between the DII score and MetS (adjusted OR_DII quartile 4 versus 1_ = 1.39, 95% CI: 1.01–1.92) after a 13-year follow-up [31]. In another prospective study, the SUN cohort from Spain [32] and one cross-sectional among Lebanese adults [33], no significant association was observed. Thus, the lack of association might be partly due to the cross-sectional design or some residual confounders. In addition, possible reasons include the difference in the numbers of food parameters, the times of 24h recall and the definitions of the MetS. The possible association between DII and the incidence of CVD, as one of the major clinical outcomes in MetS, has also been examined. For example, result of the PREDIMED study showed that the multiple-adjusted hazard ratio (HR) for each additional standard deviation of the continuous DII was 1.22 (1.06–1.40), which indicated that a pro-inflammatory diet might relate with a higher risk of CVD [34].

The existence of a significant relation between MetS and inflammation has been long acknowledged. Among a series of inflammatory factors, CRP is a special liver-derived pattern-recognition molecule that contributes to host defence [35]. The abnormal increase of CRP might stimulate liver cells and monocytes to secrete more pro-inflammatory factors, which cause serine phosphorylation of insulin receptor substrate (IRS) proteins, decreasing insulin signaling, and finally increased insulin resistance from insulin-sensitive tissue [10,11]. CRP also induces adhesion molecule expression in endothelial cells, increasing the risk of CVD [36]. In this study, we did demonstrate that CRP is related to MetS and four out of five of its individual components. The same was true with other studies conducted among Chinese patients with type 2 diabetes [37] or among low-income rural residents [38]. 

According to stratified analyses by sex, the associations between DII/CRP and MetS among women are comparable to our whole sample, while these associations are limited among men. There are important and complex differences in the pathophysiology, clinical presentation, and implications on cardiovascular risk of MetS in men and women [39], which are difficult to make a consensus. Consistent with the findings of our study, Michael D. Wirth et al. also found that participants with higher DII scores were more likely to meet the diagnosis of hypertension among women (OR _Q4 versus Q1_ = 1.19, 95% CI: 1.05 to 1.34), but not men [40]. However, decreased odds of meeting the diagnosis of hypertension and MetS overall were observed for females with higher DII scores in Alexis Sokol et al.’s study [29]. When it comes to CRP, Ming-May Lai et al.’s study showed that all components of MetS are more strongly associated with CRP in women than in man and all sex interaction were significant except for hypertension, which are in line with our study [41]. In addition, the stratified analyses among men have a relative lower statistical power to detect the true effect due to the small sample size (men comprise about 34% of the overall sample), which might be another possible explanation. Further studies are needed to obtain more evidence of the possible interaction of gender.

As for DII and CRP, Shivappa et al. reported for the first time that higher DII scores (updated DII) were significantly associated with CRP (values >3 mg/L) [42]. Other studies carried out among Iranian and Korean participants and among African Americans also showed this trend [43,44,45]. In this study, the absence of an association between the DII and CRP might be explained by the reduction in the number of food parameters. To the best of our knowledge, the shortest list used to calculate the DII was 17 food parameters reported by Nitin Shivappa et al.’s study, in which inflammatory markers such as IL-6 and homocysteine were found significant associated with DII, while the CRP and fibrinogen were not [46]. Although it is not significant among the whole population, the results of stratified analyses showed that DII was associated with CRP among subjects with MetS. Coincidently, research by Ghayour-Mobarhan et al. showed no association between diet and CRP among healthy participants, while a weak association was identified among the dyslipidaemic patients [47]. Masaki Ohsawa et al.’s study showed that the association between intake of n-3PUFA and CRP was more evident in male smokers, who have higher levels of inflammatory factors than do nonsmokers [48]. Further studies should determine the possible presence of an interaction effect, which implies that the effect of DII on serum CRP varies with differing health status (illness or not). 

Another interesting finding in the present study was that participants with higher DII scores showed a higher degree of self-reported frequency of “*Shanghuo*” (heatiness). As one essential element in TCM, *Shanghuo* has been associated with some oral ailments such as oral dryness and mucosal ulcer [22], which were probably caused by consumption of a large amount of spicy food or by emotional stress such as depression, anxiety or anger [23]. A case-control study among Chinese students showed that habits such as eating barbecued food, spicy food, or insomnia would induce the occurrence of *Shanghuo* [49]. This study also demonstrates the possible association between DII, which indicates the inflammatory potential of diet, and *Shanghuo*, calling for further studies.

In order to explore the impact of changes of dietary habits, we performed subgroup analyses after removing participants who self-reported that they had changed their dietary habits. The results were similar, which indicated that the self-reported changes in dietary habits had little effect on the inflammatory potential of diet in this study. 

The primary strength of this study was its use of the DII, which was literature-derived, population-based and standardized to facilitate quantitative comparisons. To the best of our knowledge, this is the first study to report the distribution of DII scores among a relatively large, representative population of China. In addition, we do not only analyzed the association between the DII and MetS, but also explored the possible mediation effect of inflammation (CRP) between them. Further, we were able to examine the degree of self-reported frequency of “*Shanghuo*”, which contributes to further understanding and application of the DII, especially in the Chinese population.

Some limitations of our study should be mentioned. Information for some potential confounders were not collected, such as medication and family history. Additionally, we had limited information on some of the parameters of the DII, such as flavones and trans fat, which are not included in the Chinese food composition table. In addition, using only one 24 h food recall is not sufficient to give a complete overview of dietary habits, so the DII score might not be a perfect representation of the real inflammatory potential of diet. The current study only included CRP as an inflammatory marker, while recent studies have found that other inflammatory markers such as IL-6, TNF-α, adiponectin or leptin were also closely associated with MetS [9]. Unfortunately, we have no information on other possible related markers. Future studies with a prospective design and more comprehensive factors are needed to fully understand the association between the DII, inflammation, and MetS.

## 5. Conclusions

In conclusion, this study suggested a close association between CRP and MetS; however, the association between the DII and MetS was relatively limited. Given that the DII was specially associated with CRP among subjects with MetS, it may serve as a useful tool to regulate the inflammatory state.

## Figures and Tables

**Table 1:** Baseline characteristics of tertiles of the DII score, Mean ± SD or N (%).

Variables	Dietary Inflammatory Index			
	Tertile 1	Tertile 2	Tertile 3	*p* ^a^
N	566	567	579	
DII scores	−0.91(0.74)	0.62(0.30)	1.63(0.38)	
Age	53.8(15.7)	51.5(17.6)	46.2(18.0)	<0.001 *
BMI (kg/m^2^)	24.0(3.6)	23.8(3.4)	23.6(3.6)	0.157
**Gender**				0.047 *
Male	173(30.6)	192(33.9)	217(37.5)	
Female	393(69.4)	375(66.1)	362(62.5)	
**Education**				0.218
Never	28(4.9)	16(2.8)	30(5.2)	
Secondary or under	155(27.4)	176(31.2)	160(27.8)	
high or equal	242(42.8)	238(42.1)	222(38.6)	
Bachelor	118(20.8)	108(19.1)	136(23.7)	
Master or above	23(4.1)	27(4.8)	27(4.7)	
**Family monthly income** (RMB: yuan)				0.105
≤3000	113(20.1)	114(20.2)	134(23.3)	
3000–4999	144(25.6)	169(30.0)	176(30.6)	
5000–7999	138(24.5)	106(18.8)	122(21.2)	
8000–9999	61(10.8)	54(9.6)	57 (9.9)	
10,000–14,999	58 (10.3)	66(11.7)	48(8.3)	
≥15,000	49(8.7)	55(9.8)	39(6.8)	
**Family monthly expenditure on food** (RMB: yuan)				<0.001 *
≤500	30(5.3)	36(6.4)	52(9.0)	
500–999	112(19.8)	138(24.4)	143(24.8)	
1000–2999	288(50.9)	288(50.9)	302(52.3)	
3000–4999	104(18.4)	68(12.0)	65(11.3)	
≥5000	32(5.7)	36(6.4)	15(2.6)	
**City**				0.002 *
Beijing	120(21.2)	105(18.5)	101(17.4)	
Chengdu	53(9.4)	51(9.0)	64(11.1)	
Chenzhou	57(10.1)	45(7.9)	71(12.3)	
Jilin	33(5.8)	69(12.2)	63(10.9)	
Guangzhou	122(21.6)	107(18.9)	97(16.8)	
Lanzhou	64(11.3)	60(10.6)	51(8.8)	
Wuhu	59(10.4)	58(10.2)	79(13.6)	
Xuchang	58(10.3)	72(12.7)	53(9.2)	
**Geographic location**				0.029 *
South	291(51.4)	261(46.0)	311(53.7)	
North	275(48.6)	306(54.0)	268(46.3)	
**Physical activity**				0.499
Low	109(20.3)	100(19.3)	119(21.5)	
Medium	301(56.2)	291(56.3)	286(51.6)	
High	126(23.5)	126(24.4)	149(26.9)	
**Smoking status**				0.102
Non-smoker	436(77.7)	431(76.6)	431(74.7)	
Former smoker	68(12.1)	52(9.2)	62(10.8)	
Smoker	57(10.2)	80(14.2)	84(14.6)	

DII: dietary inflammatory index, SD: standard deviation, BMI: body mass index, RMB: RenMinbi. ^a^ Age and BMI were continuous variables, and they were analyzed with one-way ANOVA test; Other variables were analyzed with chi-square analysis. * Significant *p* values that met the 5% level.

**Table 2:** Nutrients intakes of tertiles of the DII, P50th (P25th, P75th) ^a^.

Nutrients	Dietary Inflammatory Index			
	Tertile 1	Tertile 2	Tertile 3	*p* ^b^
Carbohydrate (g)	280.8(239.0,323.5)	288.2(241.9,326.8)	272.6(221.7,318.6)	0.003 *
Protein (g)	73.2(62.4,84.8)	65.2(56.3,76.4)	57.8(49.6,67.5)	<0.001 *
Total fat (g)	72.7(54.8,89.2)	69(53.7,86.3)	76(57.8,96.7)	<0.001 *
Saturated fat (g)	10.4(6.7,14.5)	10.2(6.9,14.5)	10.1(6.7,16.2)	0.674
MUFA (g)	19.8(13,27.5)	18.9(11.8,28.6)	21(13.6,32.2)	0.010 *
PUFA (g)	15.4(10.2,22.2)	13.9(9.9,18.7)	11.6(8.2,16.2)	<0.001 *
Fiber (g)	17.7(13.3,24.4)	11(8.6,13.8)	7.3(5.7,9.5)	<0.001 *
Cholesterol (mg)	341.1(107.5,600.0)	304.5(111.2,625.6)	349.3(122.3,564.5)	0.911
Niacin (mg)	15.4(12.3,19.5)	13.3(10.9,16.6)	11.4(9.3,14.0)	<0.001 *
Thiamine (mg)	1.1(0.9,1.3)	1.0(0.8,1.2)	0.9(0.7,1.0)	<0.001 *
Riboflavin (mg)	1.1(0.9,1.5)	0.9(0.7,1.2)	0.7(0.6,1.0)	<0.001 *
Folic acid (μg)	349.9(243.5,469.6)	230.9(165.9,300.2)	154.7(116.7,196.0)	<0.001 *
Vitamins A (RE)	635.2(388.1,1054.7)	379.9(222.6,574.2)	278.8(169.4,408.6)	<0.001 *
Vitamins B6 (mg)	1.3(1.1,1.6)	1.0(0.8,1.2)	0.8(0.7,1.0)	<0.001 *
Vitamins B12 (μg)	2.3(0.8,5.1)	2.2(0.8,4.7)	1.9(0.9,3.5)	0.011 *
Vitamins C (mg)	141.8(85.6,198.2)	74.8(41.3,109.9)	39.4(20.9,60.9)	<0.001 *
Vitamins E (mg)	33.1(23.7,43.8)	25.1(17.6,37.0)	17.8(12.3,30.5)	<0.001 *
Iron (mg)	24.9(21.7,30.3)	20.5(18.2,23.4)	17.4(15.4,19.5)	<0.001 *
Magnesium (mg)	368.2(322.8,440.2)	288.5(258.5,327.7)	226.4(194.3,262.8)	<0.001 *
Selenium (μg)	44.6(33.7,59.2)	41.9(31.3,55.4)	38.8(29.9,48.8)	<0.001 *
Zinc (mg)	13.1(11.7,14.9)	11.4(10.3,12.8)	10.0(9.1,11.3)	<0.001 *

DII: dietary inflammatory index, MUFA: monounsaturated fatty acids, PUFA: polyunsaturated fatty acids, RE: retinol equivalent. ^a^ Nutrients data after adjustment with nutrient density method; ^b^ Distributions of nutrients intakes were non-normal, presented as P50th (P25th, P75th), and analyzed with Kruskal–Wallis tests; * Significant *p* values that met the 5% level.

**Table 3:** Association between the incidence of MetS (components) and DII/CRP ^a^.

MetS/Components	Dietary Inflammatory Index OR (95% CI)				CRP (Continuous) OR (95% CI)
	Tertile 1	Tertile 2	Tertile 3	Continuous	
**Metabolic Syndrome**					
Model 1 ^b^	1.00(Ref.)	0.74(0.58–0.95)	0.72(0.56–0.92)	0.85(0.78–0.92)	3.83(3.05–4.80)
Model 2 ^c^	1.00(Ref.)	0.76(0.56–1.03)	1.02(0.75–1.40)	0.93(0.83–1.04)	1.66(1.26–2.18) *
**Waist circumference**					
Model 1 ^b^	1.00(Ref.)	0.86(0.68–1.09)	0.69(0.54–0.86)	0.85(0.79–0.93)	4.61(3.67–5.79)
Model 2 ^c^	1.00(Ref.)	0.92(0.64–1.32)	0.86(0.59–1.24)	0.93(0.81–1.06)	1.91(1.36–2.66) *
**Blood pressure**					
Model 1 ^b^	1.00(Ref.)	0.96(0.76–1.21)	0.86(0.68–1.08)	0.91(0.84–0.99)	2.80(2.28–3.45)
Model 2 ^c^	1.00(Ref.)	1.04(0.77–1.39)	1.40(1.03–1.89) *	1.06(0.96–1.18)	1.17(0.89–1.53)
**HDL-cholesterol**					
Model 1 ^b^	1.00(Ref.)	0.90(0.69–1.17)	1.01(0.78–1.31)	0.96(0.88–1.06)	2.13(1.72–2.64)
Model 2 ^c^	1.00(Ref.)	0.95(0.71–1.26)	1.17(0.88–1.56)	1.02(0.92–1.12)	1.64(1.27–2.11) *
**Triglycerides**					
Model 1 ^b^	1.00(Ref.)	0.69(0.54–0.88)	0.73(0.58–0.93)	0.88(0.81–0.96)	3.01(2.43–3.72)
Model 2 ^c^	1.00(Ref.)	0.73(0.55–0.96)	1.03(0.78–1.37)	0.99(0.90–1.09)	1.44(1.12–1.85) *
**Fasting glucose**					
Model 1 ^b^	1.00(Ref.)	0.78(0.61–1.00)	0.71(0.56–0.92)	0.86(0.79–0.94)	2.47(2.00–3.05)
Model 2 ^c^	1.00(Ref.)	0.78(0.59–1.04)	0.85(0.64–1.14)	0.91(0.82–1.00)	1.73(1.34–2.24) *

MetS: metabolic syndrome, DII: dietary inflammatory index, CRP: C-reactive protein, HDL-cholesterol: high-density lipoprotein cholesterol. ^a^ MetS outcomes and its components were analyzed as dichotomous variables with binary logistic regression; ^b^ Model 1 was used to obtain the crude odds ratio; ^c^ Model 2 was adjusted for age, gender, city, education level, family monthly expenditure on food, smoking status and BMI. * OR (95% CI) of adjusted model that were significant.

**Table 4:** Stratified analysis of association between the incidence of MetS (components) and DII/CRP ^a^ by sex.

MetS/Components	Dietary Inflammatory Index OR (95% CI)				CRP (Continuous) OR (95% CI)
	Tertile 1	Tertile 2	Tertile 3	Continuous	
**Males**					
**Metabolic Syndrome**					
Model 1 ^b^	1.00(Ref.)	0.86(0.56–1.32)	0.86(0.57–1.30)	0.92(0.80–1.07)	1.67(1.15–2.41)
Model 2 ^c^	1.00(Ref.)	0.91(0.55–1.50)	1.06(0.64–1.75)	0.97(0.82–1.16)	1.09(0.70–1.70)
**Waist circumference**					
Model 1 ^b^	1.00(Ref.)	1.03(0.68–1.57)	0.97(0.65–1.46)	0.95(0.82–1.09)	2.63(1.79–3.87)
Model 2 ^c^	1.00(Ref.)	1.07(0.59–1.96)	1.21(0.66–2.23)	0.99(0.80–1.23)	1.73(0.97–3.08)
**Blood pressure**					
Model 1 ^b^	1.00(Ref.)	1.08(0.70–1.66)	0.72(0.48–1.08)	0.87(0.75–1.01)	1.45(1.00–2.09)
Model 2 ^c^	1.00(Ref.)	1.25(0.76–2.07)	1.04(0.63–1.70)	0.99(0.83–1.17)	0.97(0.62–1.53)
**HDL-Cholesterol**					
Model 1 ^b^	1.00(Ref.)	1.09(0.64–1.86)	1.40(0.84–2.31)	1.08(0.91–1.30)	1.65(1.07–2.53)
Model 2 ^c^	1.00(Ref.)	1.16(0.65–2.07)	1.47(0.83–2.58)	1.10(0.90–1.35)	1.31(0.80–2.17)
**Triglycerides**					
Model 1 ^b^	1.00(Ref.)	0.77(0.50–1.17)	0.76(0.50–1.13)	0.93(0.81–1.08)	1.52(1.06–2.18)
Model 2 ^c^	1.00(Ref.)	0.75(0.47–1.19)	0.78(0.49–1.20)	0.94(0.80–1.11)	0.92(0.61–1.39)
**Fasting glucose**					
Model 1 ^b^	1.00(Ref.)	0.88(0.58–1.33)	0.67(0.45–1.01)	0.85(0.75–0.94)	1.81(1.26–2.61)
Model 2 ^c^	1.00(Ref.)	0.96(0.60–1.54)	0.90(0.56–1.44)	0.91(0.77–1.08)	1.79(1.17–2.73) *
**Females**					
**Metabolic Syndrome**					
Model 1 ^b^	1.00(Ref.)	0.69(0.51–0.93)	0.65(0.48–0.87)	0.80(0.72–0.89)	5.96(4.43–8.02)
Model 2 ^c^	1.00(Ref.)	0.67(0.45–1.00)	1.01(0.66–1.53)	0.90(0.78–1.04)	2.17(1.49–3.15) *
**Waist circumference**					
Model 1 ^b^	1.00(Ref.)	0.81(0.61–1.07)	0.59(0.44–0.79)	0.82(0.74–0.91)	6.70(4.99–9.00)
Model 2 ^c^	1.00(Ref.)	0.91(0.56–1.46)	0.74(0.45–1.20)	0.92(0.77–1.08)	1.85(1.20–2.88) *
**Blood pressure**					
Model 1 ^b^	1.00(Ref.)	0.87(0.65–1.16)	0.86(0.64–1.15)	0.90(0.81–0.99)	3.53(2.72–4.58)
Model 2 ^c^	1.00(Ref.)	0.90(0.62–1.32)	1.72(1.15–2.56) *	1.12(0.98–1.28)	1.25(0.88–1.77)
**HDL-Cholesterol**					
Model 1 ^b^	1.00(Ref.)	0.86(0.63–1.17)	0.94(0.69–1.28)	0.94(0.84–1.05)	2.50(1.94–3.23)
Model 2 ^c^	1.00(Ref.)	0.90(0.65–1.25)	1.06(0.76–1.49)	0.99(0.88–1.11)	1.77(1.31–2.38) *
**Triglycerides**					
Model 1 ^b^	1.00(Ref.)	0.64(0.47–0.86)	0.70(0.52–0.94)	0.84(0.76–0.94)	4.15(3.16–5.45)
Model 2 ^c^	1.00(Ref.)	0.66(0.46–0.94) *	1.17(0.80–1.70)	1.01(0.89–1.15)	1.86(1.33–2.60) *
**Fasting glucose**					
Model 1 ^b^	1.00(Ref.)	0.69(0.51–0.95)	0.69(0.50–0.94)	0.84(0.75–0.94)	2.77(2.13–3.62)
Model 2 ^c^	1.00(Ref.)	0.68(0.47–0.98) *	0.83(0.57–1.21)	0.89(0.78–1.02)	1.69(1.21–2.37) *

MetS: metabolic syndrome, DII: dietary inflammatory index, CRP: C-reactive protein, HDL-cholesterol: high-density lipoprotein cholesterol. ^a^ MetS outcomes and its components were analyzed as dichotomous variables with binary logistic regression; ^b^ Model 1 was used to obtain the crude odds ratio; ^c^ Model 2 was adjusted for age, city, education level, family monthly expenditure on food, smoking status and BMI. * OR (95% CI) of adjusted model that were significant.

**Table 5:** Association between the DII and CRP ^a^.

Subjects	Dietary Inflammatory Beta Estimates (95% CI)			
	Tertile 1	Tertile 2	Tertile 3	Continuous
**Whole sample**				
Model 1 ^b^	1.00(Ref.)	−0.004(−0.064,0.055)	−0.032(−0.101,0.036)	−0.012(−0.033,0.009)
Model 2 ^c^	1.00(Ref.)	0.035(−0.018,0.089)	0.040(−0.024,0.103)	0.012(−0.008,0.031)
**Subjects with MetS**				
Model 1 ^b^	1.00(Ref.)	0.072(−0.010,0.154)	0.110(0.009,0.211)	0.033(0.003,0.062)
Model 2 ^c^	1.00(Ref.)	0.086(0.004,0.167) *	0.145(0.045,0.245) *	0.040(0.010,0.069) *
**Subjects without MetS**				
Model 1 ^b^	1.00(Ref.)	−0.021(−0.097,0.056)	−0.048(−0.134,0.038)	−0.018(−0.045,0.009)
Model 2 ^c^	1.00(Ref.)	0.021(−0.050,0.091)	0.001(−0.080,0.082)	0.002(−0.024,0.027)

DII: dietary inflammatory index, CRP: C-reactive protein, MetS: metabolic syndrome. ^a^ CRP was analyzed as a continuous variable with linear regression; ^b^ Model 1 was used to obtain the crude beta estimates; ^c^ Model 2 was adjusted for age, gender, city, education level, family monthly expenditure on food, smoking status, and BMI. * Beta estimates (95% CI) of adjusted model that were significant.

## Supplementary Materials

The following are available online at http://www.mdpi.com/2072-6643/10/7/831/s1. Table S1: Association between the incidence of MetS (components) and DII/CRP in supplementary analyses; Table S2: Stratified analysis of association between the DII and CRP by sex; Table S3: Association between the DII and CRP in supplementary analyses.
