# Supplementary material for: Association between Dietary Inflammatory Index, C-Reactive Protein and Metabolic Syndrome: A Cross-Sectional Study
Source: Nutrients. 2018 Jun 27;10(7):831. doi: 10.3390/nu10070831 (PMC6073906; doi:10.3390/nu10070831)
Supplement: Supplementary file 1 [file nutrients-10-00831-s001.pdf]

Table S1. Association between the incidence of MetS (components) and DII/CRP in supplementary analyses [a]

MetS/components	Dietary inflammatory index OR(95% CI)				CRP(Continuous) OR(95% CI)
	Tertile 1	Tertile 2	Tertile 3	Continuous	
**Metabolic Syndrome**					
Model 1[b]	1.00(Ref.)	0.81(0.61–1.07)	0.70(0.53–0.93)	0.87(0.79–0.96)	4.51(3.48–5.85)
Model 2[c]	1.00(Ref.)	0.93(0.65–1.31)	1.05(0.73–1.52)	0.99(0.87–1.12)	1.80(1.31–2.47)*
**Waist circumference**					
Model 1[b]	1.00(Ref.)	0.84(0.65–1.10)	0.66(0.51–0.87)	0.86(0.78–0.94)	4.61(3.67–5.79)
Model 2[c]	1.00(Ref.)	0.94(0.63–1.42)	0.90(0.59–1.36)	0.94(0.81–1.09)	1.95(1.34–2.83)*
**Blood pressure**					
Model 1[b]	1.00(Ref.)	0.96(0.73–1.25)	0.88(0.67–1.15)	0.93(0.84–1.02)	3.19(2.50–4.07)
Model 2[c]	1.00(Ref.)	1.09(0.78–1.52)	1.54(1.08–2.17)*	1.11(0.99–1.26)	1.41(1.03–1.92)*
**HDL-Cholesterol**					
Model 1[b]	1.00(Ref.)	0.91(0.67–1.24)	0.96(0.71–1.31)	0.97(0.87–1.08)	2.21(1.72–2.85)
Model 2[c]	1.00(Ref.)	0.96(0.69–1.34)	1.18(0.84–1.66)	1.04(0.92–1.17)	1.64(1.21–2.21)*
**Triglycerides**					
Model 1[b]	1.00(Ref.)	0.72(0.54–0.95)	0.68(0.52–0.90)	0.87(0.79–0.96)	3.22(2.51–4.12)
Model 2[c]	1.00(Ref.)	0.80(0.58–1.10)	0.99(0.71–1.38)	0.99(0.88–1.11)	1.52(1.13–2.03)*
**Fasting glucose**					
Model 1[b]	1.00(Ref.)	0.80(0.60–1.06)	0.70(0.52–0.93)	0.87(0.78–0.96)	2.65(2.10–3.38)
Model 2[c]	1.00(Ref.)	0.82(0.59–1.14)	0.84(0.60–1.19)	0.92(0.82–1.03)	1.85(1.38–2.49)*

MetS: metabolic syndrome, DII: dietary inflammatory index, CRP: C-reactive protein, HDL-cholesterol: high-density lipoprotein cholesterol. [a] MetS outcomes and its components were analyzed as dichotomous variables with binary logistic regression; [b] Model 1 was used to obtain the crude odds ratio; [c] Model 2 was adjusted for age, gender, city, education level, family monthly expenditure on food, smoking status and BMI. * OR(95%CI) of adjusted model that were significant.

Table S2. Stratified analysis of association between the DII and CRP by sex [a]

Subjects	Dietary inflammatory Beta estimates (95% CI)			
	Tertile 1	Tertile 2	Tertile 3	Continuous
**Whole sample**				
Model 1[b]	1.00(Ref.)	−0.004(−0.064,0.055)	−0.032(−0.101,0.036)	−0.012(−0.033,0.009)
Model 2[c]	1.00(Ref.)	0.035(−0.018,0.089)	0.040(−0.024,0.103)	0.012(−0.008,0.031)
**Male**				
Model 1[b]	1.00(Ref.)	−0.004(−0.112,0.082)	−0.015(−0.112,0.082)	−0.003(−0.036,0.030)
Model 2[c]	1.00(Ref.)	0.008(−0.088,0.104)	0.033(−0.075,0.140)	0.006(−0.027,0.039)
**Female**				
Model 1[b]	1.00(Ref.)	−0.003(−0.078,0.071)	−0.062(−0.150,0.026)	−0.021(−0.047,0.006)
Model 2[c]	1.00(Ref.)	0.047(−0.018,0.112)	0.046(−0.032,0.125)	0.017(−0.007,0.040)

[a] CRP was analyzed as a continuous variable with linear regression; [b] Model 1 was used to obtain the crude beta estimates; [c] Model 2 was adjusted for age, gender, city, education level, monthly cost of food consumption, smoking status, and BMI. * Significant Beta estimates (95%CI) of adjusted model.

Table S3. Association between the DII and CRP in supplementary analyses [a]

Subjects	Dietary inflammatory Beta estimates (95%CI)			
	Tertile 1	Tertile 2	Tertile 3	Continuous
**Whole sample**				
Model 1[b]	1.00(Ref.)	−0.008(−0.076,0.060)	−0.047(−0.124,0.031)	−0.011(−0.035,0.013)
Model 2[c]	1.00(Ref.)	0.031(−0.030,0.092)	0.028(−0.044,0.100)	0.012(−0.010,0.034)
**Subjects with MetS**				
Model 1[b]	1.00(Ref.)	0.100(0.004,0.197)	0.090(−0.029,0.210)	0.043(−0.0001,0.086)
Model 2[c]	1.00(Ref.)	0.089(−0.007,0.184)	0.117(−0.003,0.236)	0.040(0.005, 0.076)*
**Subjects without MetS**				
Model 1[b]	1.00(Ref.)	−0.050(−0.136,0.036)	−0.058(−0.153,0.037)	−0.022(−0.051,0.008)
Model 2[c]	1.00(Ref.)	0.007(−0.073,0.087)	−0.002(−0.093,0.088)	−0.001(−0.029,0.028)

DII: dietary inflammatory index, CRP: C-reactive protein, MetS: metabolic syndrome. [a] CRP was analyzed as a continuous variable with linear regression; [b] Model 1 was used to obtain the crude beta estimates; [c] Model 2 was adjusted for age, gender, city, education level, family monthly expenditure on food, smoking status and BMI. * Beta estimates (95%CI) of adjusted model that were significant.
